# Investigation of multimorbidity and prevalent disease combinations in older Irish adults using network analysis and association rules

**DOI:** 10.1038/s41598-019-51135-7

**Published:** 2019-10-10

**Authors:** Belinda Hernández, Richard B. Reilly, Rose Anne Kenny

**Affiliations:** 1 TILDA The Irish Longitudinal Study in Ageing, Trinity College, The University of Dublin, Dublin, Ireland; 2 Mercer Institute for Successful Ageing, St. James Hospital, Dublin, Ireland; 3 Dept of Medical Gerontology, School of Medicine, Trinity College, The University of Dublin, Dublin, Ireland; 4 School of Engineering, Trinity College, The University of Dublin, Dublin, Ireland; 5 Trinity Centre for Biomedical Engineering, Trinity College, The University of Dublin, Dublin, Ireland

**Keywords:** Comorbidities, Diseases, Geriatrics

## Abstract

Multimorbidity (the presence of multiple medical conditions) is well known to increase with age. People with multimorbidities often have higher physical and functional decline as well as increased mortality. Despite growing evidence that integrated and collaborative care improves many undesirable outcomes of multimorbidity, the majority of health systems are based around treating individual diseases. A pattern analysis of comorbidities using network graphs and a novel use of association rules was conducted to investigate disease associations on 6101 Irish adults aged 50+. The complex network of morbidities and differences in the prevalence and interactions of these morbidities by sex was also assessed. Gender specific differences in disease prevalence was found for 22/31 medical conditions included in this study. Females had a more complex network of disease associations than males with strong associations found between arthritis, osteoporosis and thyroid issues among others. To assess the strength of these associations we provide probabilities of being diagnosed with a comorbid condition given the presence of an index morbidity for 639 pairwise combinations. This information can be used to guide clinicians in deciding which comorbidities should be incorporated into comprehensive assessments in addition to anticipating likely future morbidities and thus developing prevention strategies.

## Introduction

Population ageing is a worldwide phenomenon. The United Nations estimates that globally the number of people aged 60+ is expected to more than double by 2050 and to more than triple by 2100 (from 962 million in 2017 to 3.1 billion in 2100)^1^. The Irish population, in line with global trends is also rapidly ageing and becoming more age dependent (where age dependency is measured as the ratio of 0–14 and 65+ compared to 15–64 year olds). In 2016 37.2% of the Irish population were aged 45 or older; an increase of 9.6% in the 30 years previous^2^. Although this increase in average life span and change in global age structure towards a rapidly ageing society is a major success for medical and public health systems, it also represents new challenges for public health policy to ensure that *healthy* life expectancy is increased rather than just life expectancy^3^.

Multimorbidity is defined as the co-existence of two or more chronic conditions, each one of which is either: (1) A physical non-communicable disease of long duration, such as a cardiovascular disease or cancer. (2) A mental health condition of long duration, such as a mood disorder or dementia. (3) An infectious disease of long duration, such as HIV or hepatitis C. Multimorbidity is further defined as the co-existence of several conditions where none are considered an index condition that is the specific focus of attention^4–6^. It is this definition of multimorbidity which we use in the following work.

The issue of multimorbidity is a common one and is well known to increase with age^7,8^. The majority of older adults suffer from multimorbidities and the presence of multimorbidities has also been shown to be highly correlated with functional and physical decline as well as mortality^9^; increased use of health care facilities^7^; decreased quality of life^10,11^ and increased health care costs^7,9,12^. The prevalence of multimorbidity is not well defined, but has been estimated to be anywhere in the region of 55–98%^13^ depending on the definition of multimorbidity; the number and type of illnesses included in the definition as well as the population being studied^7,14^.

Multimorbidity can increase the risk of geriatric syndromes^15^. With the majority of conditions being chronic in nature, the disease landscape generates unique implications for health policy and practice^16,17^. An increasing number of studies provide evidence to suggest that multimorbidity is now the norm for older adults rather than the exception^4,12,13,18,19^. However despite this growing evidence base, the majority of health care systems and public health policies are centred on the idea of treating individual diseases rather than looking at the complex network of diseases present in individual patients^7,9^. Furthermore, the majority of clinical trials and academic research which guide public policy often exclude multi-morbid individuals from the analysis and rather focus on predicting risk factors for individual diseases^20^. Treating and studying diseases in isolation may lead to duplication and inefficiencies in the case of multi-morbid patients^7^ as well as have serious implications if the treatment of one morbidity contradicts that of another^19^.

Previous studies on multimorbidity have mainly focused on the various sociodemographic and biological reasons for multimorbidity^7,11,14,21^ or on the outcomes and consequences of multimorbidity, such as its effect on quality of life, health care utility^22^ and mortality^23^ among others^24^. In recent years there has also been a shift towards identifying disease clusters and latent groupings using either ad hoc methods or more formal methods such as factor analysis or clustering algorithms^25–30^. Many studies identifying disease clusters and comorbidities have used metrics such as the observed/expected ratio (the proportion of the dataset observed with a given comorbidity divided by the product of the prevalence of the individual diseases) to identify comorbidities that occur more often than would be expected than random chance in the population^29–34^. In the datamining and association rule literature this observed/expected ratio is known as the lift of an association rule. McNicolas *et al*.^35^ in their work, pointed out a shortcoming of such a metric in that the upper and lower limits the observed/expected ratio (hereon referred to as the lift) differs depending on the overall prevalence of the individual items being studied. Using the raw lift for the analysis of comorbidities will have the effect that it will generally be biased towards low prevalence conditions (See Section 6.3 and Supp. Mat. S 2 for more details). For this reason, they developed a standardised version of the lift which takes a value between 0 and 1 and hence allows for a fair comparison when ranking the importance of disease associations in comorbidity analysis. Although association rules and observed/expected ratios have been used previously to analyse common comorbidities^36,25,27^ to the authors’ knowledge the strength of the associations have never been comprehensively and fairly compared between rules. Hence we provide a novel use of the statistical methods developed in McNicolas *et al*.^35^ to unbiasedly assess and rank the strength of the associations between diseases.

The purpose of this study is to build upon the pattern analysis literature and identify the prevalence and interactions of medical conditions in a representative sample of 6,101 older Irish adults from an Irish population study. A secondary purpose was to provide a novel use of ‘association rules’ applied to multimorbidity data in order to prune and rank important disease associations which occur more often than would be expected by random chance. We then use this subset of disease associations which occur more often than random and perform a network analysis to identify clusters of diseases and also investigate higher order disease interactions.

We argue that to make informed health care policy decisions based around appropriate intervention and prevention strategies, the pathogenesis and co-occurrence of morbidities which contribute to the identification of multi-morbid individuals must first be identified and studied. Furthermore, this information may provide insights into underlying mechanisms to explain co-occurrence. One objective of this work is to provide meaningful information for clinicians when performing comprehensive patient assessments regarding which likely multi morbidities to incorporate in evaluation when assessing an older pre-multimorbid patient. Another objective is the expectation that a better understanding of the associations between common medical conditions will inform research into the mechanisms underpinning these common disease associations.

## Results

**Figure 1:**
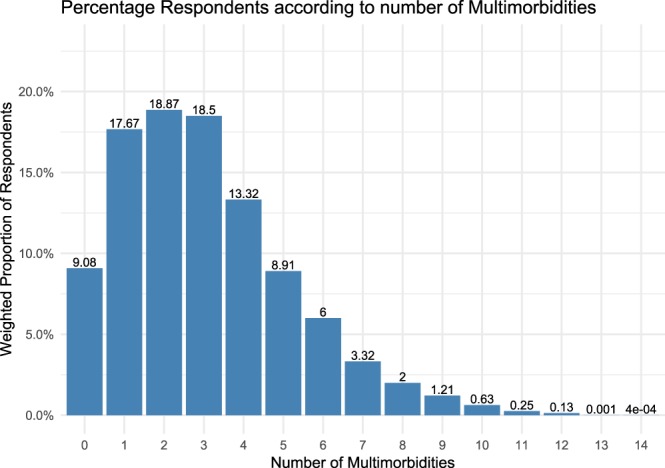
Percentage breakdown of population according to number of morbidities.

Figure 1 shows the estimated lifetime prevalence of multimorbidity for older Irish adults is 73.25% and only 9.08% of the population do not have any of the 31 diseases studied. The total number of medical conditions present in any one individual ranged from 0–14 and the median number of conditions present was 3.

Summary information on the population representative prevalence of individual morbidities as well as their prevalence and statistical significance with regards to sex can be seen in Table 1.

**Table 1:** Total disease prevalence and breakdown of prevalence by sex as a population weighted percentage.

Disease	Total % n = 6101	Male % n = 2,754 (46.26%)	Female % n = 3,347 (53.74%)	p-value
Hypertension	44.03	45.69	42.61	**0.036**
Angina	6.06	7.67	4.67	**<0.001**
Heart attack	5.08	8.13	2.45	**<0.001**
Heart failure	1.32	2.22	0.55	**<0.001**
Diabetes	9.79	12.89	7.12	**<0.001**
Stroke	2.03	2.35	1.75	0.167
TIA	3.31	3.11	3.48	0.527
High Cholesterol	50.52	48.44	52.31	**0.012**
Heart murmur	6.25	5.3	7.07	**0.016**
Heart arrhythmia	10.89	12.51	9.49	**0.001**
Cataracts	14.09	11.84	16.02	**<0.001**
Glaucoma	2.77	2.64	2.89	0.609
ARMD	2.96	2.64	3.23	0.241
Lung Disease	5.27	4.44	5.98	**0.036**
Asthma	9.6	7.56	11.35	**<0.001**
Arthritis	34.78	28.65	40.05	**<0.001**
Osteoporosis	15.92	4.85	25.45	**<0.001**
Cancer	4.73	5.23	4.3	0.151
Parkinson’s	0.69	0.91	0.51	0.112
Alcohol Abuse	2.04	3.31	0.95	**<0.001**
Ulcers	4.72	4.96	4.52	0.542
Varicose Ulcer	2.49	1.89	3	**0.024**
Liver Disease	0.69	0.75	0.64	0.687
Thyroid	8.35	2.75	13.16	**<0.001**
Kidney Disease	0.56	0.85	0.32	**0.02**
Anaemia	0.5	0.15	0.8	**0.017**
Depression	10.92	8.68	12.84	**<0.001**
Poor Hearing	2.13	2.98	1.4	**<0.001**
Poor Vision	1.96	2.1	1.85	0.55
Obesity	23.38	25.4	21.65	**0.002**
Urinary Incont.	19.3	10.44	26.93	**<0.001**

^*^TIA: transient ischemic attack; ARMD:Age Related Macular Degeneration; Urinary Incont.: Urinary Incontinence.

From Table 1, it can be seen that 22 out of the 31 medical conditions included in this study were significantly related to sex. The majority of cardiovascular diseases and related comorbidities such as diabetes and obesity are highly over-represented by males. Multi morbid associations for angina, heart attacks, heart failure, irregular heart rhythms, diabetes, obesity, alcohol abuse, kidney disease and poor hearing are all significantly more prevalent in men (Table 1). Also, osteoporosis, arthritis, urinary incontinence and thyroid illnesses are over represented by females (Table 1).

### Finding comorbidities occurring more often than random

**Figure 2:**
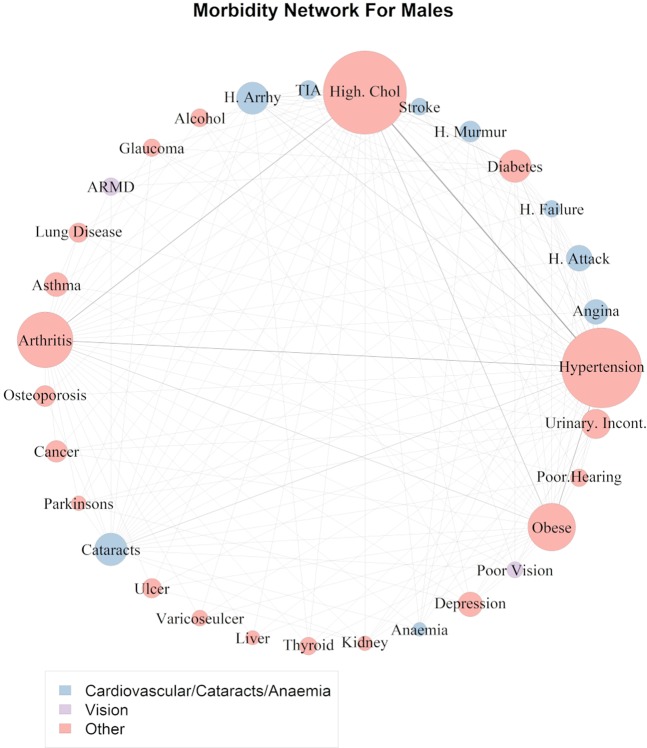
Network of co-morbidities for males. The size of the nodes (circle containing the morbidity name) is proportional to the overall prevalence of the disease. The edge width connecting the vertices is proportional to the number of participants with the comorbidity indicated by both vertices.

**Figure 3:**
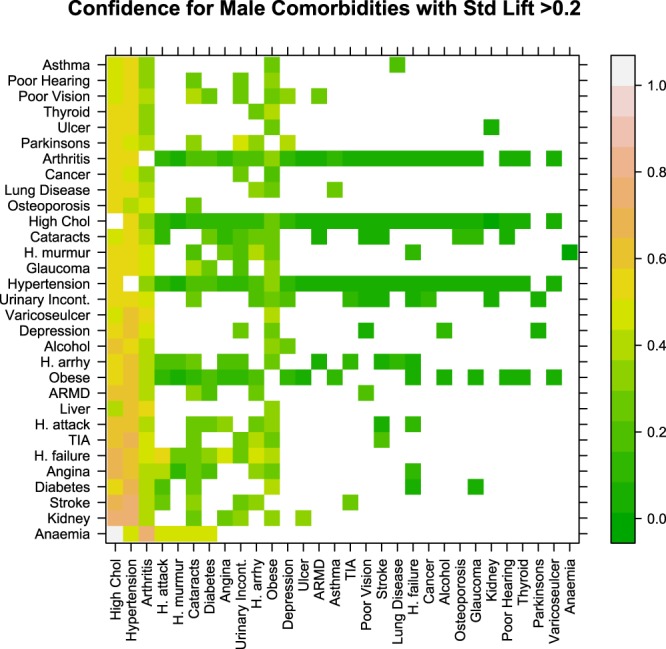
Confidence measure for male comorbidities with a standardised lift value >0.2. The colour of the grid points indicate the probability of having the disease indicated by the x-axis given that the disease indicated on the y-axis is already present, the lighter the colour the higher the probability. Take as an example the grid point in the top left hand corner of Fig. 3, this indicates that the probability of having high cholesterol given asthma is already present in males is 0.478.

To investigate the set of comorbidities identified as occurring more often than random chance; a network of pairwise disease associations was developed to visualise the prevalence of the comorbidities identified as having a standardised lift >0.2. A fast-greedy network community detection algorithm was also used to identify connected communities/clusters of diseases for the male and female cohorts (see Section 6 for further details). Figure 2 shows the network graph linking comorbidities which have a standardised lift of >0.2 for the male cohort and Fig. 3 shows the confidence measurements with a standardised lift >0.2 for the male cohort. Confidence measures for all 961 pairwise combinations of morbidities for males and females can be seen in Supp. Mat. Figs S.3.1 and S.3.2.

Figure 2 shows that the most common co-morbidities in males involve connections between high cholesterol, hypertension, obesity, cataracts and arthritis. From Fig. 3 it can also be seen that hypertension, high cholesterol, arthritis and obesity have the highest number of associations with other medical conditions in males.

Hypertension and high cholesterol was the most common comorbidity which affected 26.98% of all males (Fig. 2). Of the males who were hypertensive 57.6% also had high cholesterol (Fig. 3) and these comorbidities occurred together 17% more often that would be expected if they were randomly associated with each other (standardised lift 0.57). Hypertension and arthritis (prevalence 16.16%), high cholesterol and arthritis (prevalence 15.5%), obesity and hypertension (prevalence 14.60%), obesity and high cholesterol (prevalence 12.45%) followed by diabetes and hypertension (prevalence 9.15%) and obesity and arthritis (prevalence 8.86%) were the highest prevalence comorbidities in the male cohort (Fig. 2). From Fig. 3, it can be seen, that of the males with arthritis: 54.14% were also hypertensive; 51.95% had high cholesterol and 29.68% were obese. Furthermore, of those who were obese; 60.63% were hypertensive; 51.73% had high cholesterol and 36.8% had arthritis (Fig. 3).

**Figure 4:**
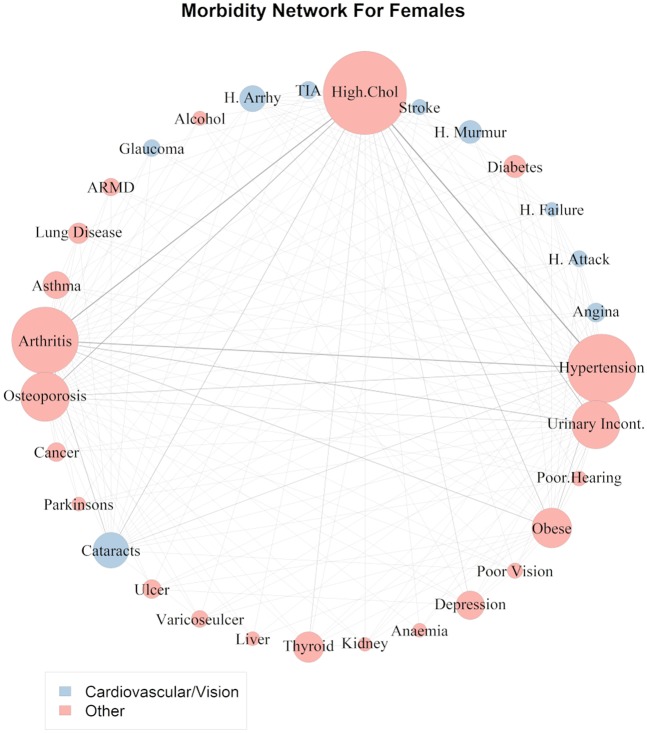
Network of comorbidities for females. The size of the nodes (circle containing the morbidity name) is proportional to the overall prevalence of the disease. The edge width connecting the vertices is proportional to the number of participants with the comorbidity indicated by both vertices.

**Figure 5:**
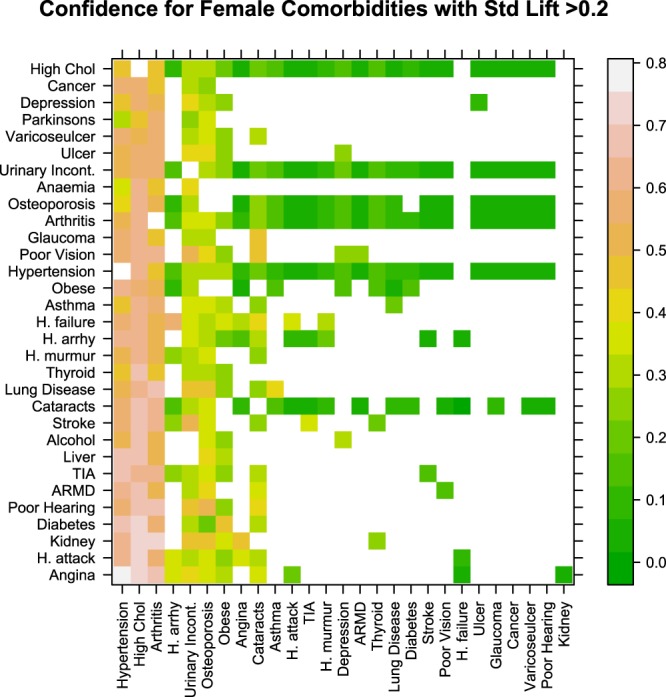
Confidence measure for female comorbidities with a standardised lift value >0.2. The colour of the grid points indicate the probability of having the disease indicated by the x-axis given that the disease indicated on the y-axis is already present, the lighter the colour the higher the probability. Take as an example the grid point in the top left hand corner of Fig. 5, this indicates that the probability of having hypertension given high cholesterol is already present in females is 0.485.

Figure 4 shows the female disease network plot of comorbidities with standardised lift >0.2 and Fig. 5 shows confidence measurements for this subset of rules for the female cohort.

From Fig. 4 it can be seen that there is a much more complex set of highly occurring associated conditions in the female cohort than the male cohort (Fig. 2). For example, there are strong links between high cholesterol and hypertension, obesity, arthritis, osteoporosis, cataracts and urinary incontinence. Figure 5 shows that like the male cohort, hypertension, high cholesterol and arthritis also have the highest number of associations with other medical conditions in females. It can also be seen that females have a high probability of suffering from osteoporosis, urinary incontinence, obesity and cataracts given the presence of a wide range of conditions.

As in the male cohort, hypertension and high cholesterol was also the highest prevalence comorbidity in Irish females with a prevalence of 26.2% (standardised lift 0.61) (Fig. 4). Of those who were hypertensive; 61.16% also had high cholesterol where as 48.51% of those with high cholesterol were hypertensive (Fig. 5). Arthritis and high cholesterol (prevalence 24.95%), arthritis and hypertension (prevalence 21%), osteoporosis and high cholesterol (prevalence 16.67%), urinary incontinence and high cholesterol (prevalence 16.01%), urinary incontinence and arthritis (prevalence 14.88%) followed by osteoporosis and arthritis (prevalence 13.8%) were the most prevalent comorbidities in females (Fig. 4). Of those females with arthritis: 60.51% had high cholesterol, 50.9% were hypertensive 36.09% had urinary incontinence and 33.48% had osteoporosis (Fig. 5). Of the females with osteoporosis: 61.13% had high cholesterol, 49.87% had arthritis, 43.53% were hypertensive and 32% had urinary incontinence. Of those with urinary incontinence: 59.49% were hypertensive, 55.27% had arthritis, 49.6% were hypertensive, 32.96% had osteoporosis and 24.97% were obese (Fig. 5).

### Uncovering disease clusters

From the network of pairwise disease associations for each of the male and female cohorts (Figs 2 and 4) the “fast greedy” community detection algorithm was implemented to uncover clusters of diseases present in the male and female networks respectively. The colour of the disease nodes in Figs 2 and 4 show the disease networks identified. The labels for these clusters were derived manually from investigation of the diseases present in each group. The detection algorithm uncovered three disease clusters for the male cohort (Fig. 2):

- Cardiovascular/Cataracts/Anaemia
- Vision
- Other

The cardiovasulcar/ cataracts/ anaemia group consisted of heart arrythmia, TIA, stroke, heart murmur, heart failure, heart attack, angina, anaemia and cataracts. The vision group separated poor vision and age-related macular degeneration from the other diseases and the third group labelled “other” contained all the remaining conditions. In the female cohort there were two main clusters of conditions identified:

- Cardiovascular/Vision
- Other

The cardiovascular/vision group was quite similar to the male grouping and consisted of heart arrythmia, TIA, stroke, heart murmur, heart failure, heart attack, angina, cataracts and glaucoma (Fig. 4).

### Higher order interactions of diseases

Groups of unique interactions between three diseases (triplets) for the male and female cohorts with standardised lift >0.2 were also identified. Looking at unique triplets with standardised lift >0.2, it was found that for the male cohort a total of 2735 unique triplets exist; 838 of which only occur in one person. Only 3 triads have prevalence >5% and are shown in Table 2.

**Table 2:** Unique triads of medical conditions in males with prevalence >5%.

Medical Conditions	Prevalence
High Cholesterol, Hypertension, Arthritis	9.77%
High Cholesterol, Hypertension, Obesity	8.82%
Hypertension, Obesity, Arthritis	5.95%

For the female cohort 2388 unique triads of diseases were uncovered; 796 of which only occur in one person. Only 16 of these disease combinations occur with prevalence higher than 5% (shown in Table 3).

**Table 3:** Triads of medical conditions in females with prevalence >5%.

Medical Conditions	Prevalence
High Cholesterol, Arthritis, Hypertension	13.92%
High Cholesterol, Arthritis, Urinary Incontinence	9.38%
High Cholesterol, Arthritis, Osteoporosis	8.78%
High Cholesterol, Hypertension, Urinary Incontinence	8.69%
Hypertension, Arthritis, Urinary Incontinence	8.34%
High Cholesterol, Hypertension Osteoporosis	8.1%
Hypertension, High Cholesterol, Obesity	7.32%
High Cholesterol, Arthritis, Cataracts	7.29%
Hypertension, Arthritis, Osteoporosis	6.75%
Hypertension, Arthritis, Obesity	6.69%
Hypertension, High Cholesterol Cataracts	6.57%
Hypertension, Arthritis, Cataracts	6.33%
High Cholesterol, Arthritis, Obesity	6.01%
High Cholesterol, Osteoporosis, Urinary Incontinence	5.92%
Arthritis, Urinary Incontinence, Osteoporosis	5.53%

## Discussion

The analysis presented supports evidence that multimorbidity is now the norm rather than the exception for ageing adults^19^. We found that the population weighted prevalence of multi-morbidity in our population representative sample of 6,101 Irish adults aged 50+ was 73.25% which confirms the assertion that multi-morbidity in older adults is a problem with high prevalence and a serious issue that needs to be addressed. We also provide probabilities of being diagnosed with a comorbid condition given the presence of another morbidity cross-sectionally for a total of 639 pairwise combinations of morbidities (309 male, 330 female) which co-occurred more often than expected by random chance. This information can be used to guide clinicians in determining the risk of individual comorbidities when presented with patients who are pre multi-morbid and also help them in determining an overall intervention and prevention strategy on an individual level. The clinical utility of such information is strong and can be tailored depending on the specific condition(s) under scrutiny. For example, we showed evidence to suggest that during clinical assessments elderly patients who present with high cholesterol and hypertension should also be assessed for diabetes and arthritis and possibly osteoporosis if female which is not currently standard practice in a clinical geriatric assessment. The fact that nearly one third of all females with arthritis (33.48%), high cholesterol (29.6%), osteoporosis (32%) and hypertension (31.17%) also experienced urinary incontinence suggests that incontinence should also be assessed in the presence of these conditions in females. Urinary incontinence is often an under diagnosed issue and it has previously been reported that at least one-third of older adults with urinary incontinence do not report it and so remain untreated^37,38^. It is also not common practice for practitioners to ask about incontinence unprompted. The psychological and social effects of urinary incontinence in older adults has also been discussed in detail in the literature, and it has been found to be associated with social isolation, depression and anxiety^37–39^.

As gender effects for multi morbidities have previously been shown to be significant^12,18^ and would clinically be expected for some morbidities for example in osteoporosis and urinary incontinence^38^; it was decided to investigate the prevalence of individual morbidities with regards to gender. The current study found evidence to support these findings as 22 of the 31 conditions considered were found to be statistically significantly different with regard to sex. Previous studies have also reported higher risk and higher number of multi-morbid conditions for females compared to males^4,40,41^ which is in line with the findings of this study.

Clustering of disease associations present in the pairwise network of male and female comorbidities uncovered two main disease clusters for females and three for males. In the case of Irish females, cataracts, glaucoma and all cardiovascular diseases were assigned to the same cluster indicating that associations between these conditions are more similar to each other than to conditions in other groups. The male comorbidity network uncovered a similar finding and identified a group which consisted of cardiovascular conditions, cataracts and chronic anaemia. The links between cataracts and cardiovascular disease in both sexes have been well established in the literature and it has long been noted that cataracts and many cardiovascular diseases share common risk factors such as diabetes, hypertension and obesity^42–44^. Our analysis showed that ~23% of all men with heart attack, heart failure, heart murmur, TIA and heart arrhythmias; 25.8% of those with angina and 30.3% of those with stroke also have cataracts (Fig. 3). These findings were even more pronounced in females (Fig. 5). This suggests that patients with cardiovascular disease should also be referred to an ophthalmologist or other eye specialist to screen for cataracts. The role of anaemia in heart failure and cardiovascular disease (especially in the presence of kidney disease) has also been well established^45,46^. Sarnak *et al*. also found that anaemia was associated with increased risk of cardiovascular disease for the general population with and without kidney disease in a US cohort of 15,792 subjects aged 45–64^47^. Although our study had very low prevalence of chronic anaemia (only 4 respondents in the male cohort) the fact that anaemia was clustered together with the cardiovascular diseases lends some support to these findings.

Looking at the complex network of morbidities present in the current study, it is clear that the combinations and number of morbidities suffered is very heterogeneous. This means that the care strategy taken for management of these complex sets of conditions needs to be made at an individual level. The importance of looking at the entirety of the patient’s physical and mental health rather than treating symptoms for individual morbidities is also clear and well documented^7,48–50^ As clinical practices and guidelines are generally aimed at the treatment of individual diseases; multimorbid patients tend to suffer a greater drug burden and risk of complications due to incongruent guidelines from treating each morbidity individually^7^. For example, associations between hypertension and arthritis are commonly associated with age and have been shown in this study to be the among the most prevalent disease associations in both sexes. It is also known that the use of non-steroidal anti-inflammatory drugs (NSAIDs) (commonly used to treat pain in inflammatory conditions such as arthritis) decrease the effectiveness of antihypertensive drugs and have also been shown to cause renal injury and dysfunction^51,52^. Therefore, ignoring the overall medical history and comorbidities of a patient could have grave implications for their overall health, especially when drug interactions are present. Furthermore, patients with multimorbidity are often excluded from clinical trials which form the evidence base that determine recommended treatment strategies for individual conditions^53^. Given the high prevalence of multimorbidity, such exclusions potentially obviate the generalisability of such trials.

The effective treatment of multimorbidity in health care systems is particularly challenging as often there are no IT systems in place to provide patients with a unique identifier to track and share patients’ health records between the various individual primary and secondary health care systems. This means that specialist care givers may not be aware of other co-existing conditions when treating individual illnesses and as a result care and communication between the various specialists may be fragmented. The need for integrated care and individualised care plans where one clinician or gerontologist is responsible for coordinating the care of each individual morbidity has been well documented^54–56^ and the complex interrelationship between morbidities in ageing Irish adults shown in this work supports this evidence.

### Limitations

One of the major limitations in the study of multimorbidities and in the comparison of results across different multimorbidity studies in general is differences in the list of medical conditions included and in how the data was collected i.e. self-reported versus clinically diagnosed conditions. Here we included all 31 medical conditions which are collected as part of the TILDA study. Ireland currently does not have a fully linked electronic health record system and so data linkage to medical records was not an option in this case. Had direct access to medical records been possible however, the patterns and prevalence of detected patterns and disease associations may have been different to the self-reported conditions we presented in this work. Self-reported diagnoses have previously been criticised^57^ as they rely on a number of subjective factors such as the participants’ memory and understanding of diagnoses (for example for chronic diseases participants may think they are cured of a condition when in fact the condition is under control). Also, at times there may be a mismatch in the understanding of language used by health care professionals such as say high blood sugar implying diabetes or indeed participants may perceive they have a condition without any actual medical diagnosis. However, a number of studies have found that self-reported measurement of multimorbidity have high concordance with data based on medical diagnoses^58–60^. The TILDA questionnaire tries as much as possible to address these issues by specifically asking if a doctor has diagnosed any of the given conditions and also includes alternative phrases for medical conditions using non-medical terminology.

Another limitation in accessing the true underlying prevalence of multimorbidity and its resulting disease patterns is that participants have to engage with the health care system in order to be diagnosed, therefore a number of conditions may be under-diagnosed and hence under-reported especially for those sections of the population where there are barriers to entry to the health care system (for example lower SES respondents who do not qualify for free medical care).

With regards to the statistical methods used we have attempted as much as possible to select the sets of associations which are occurring more often than would be expected by random chance and to correct for bias in the observed/expected ratio which is commonly used in the multimorbidity literature. The set of associations presented in this article were selected using a threshold value of 0.2 for the standardised lift. As with any threshold or cut off value (such as selecting p-value < 0.05 to imply a statistically significant result) the results may vary depending on the threshold value chosen. To alleviate this the authors did experiment with various values of the threshold and performed an ad hoc sensitivity analysis to this value. In general, it was found that results were reasonably stable to the threshold chosen. Smaller values of the threshold obviously allowed more associations to be selected and larger threshold values unsurprisingly shortened the list of values included. However, the main results presented in this article remained unchanged for a wide range of threshold values. It should also be noted that the value of the standardised lift/observed-expected ratio depends on the lower limit placed on the support and confidence of the analysis. In this work, no lower limit threshold was placed on these measures and all disease interactions were included in the analysis. The work presented in this article is an investigation into co-occurrences of diseases and the prevalence of these disease interactions. Future work will focus on investigating risk factors for specific combinations of diseases; performing a population representative analysis of disease interactions and investigating emerging, persistent and decreasing trends in disease interactions longitudinally.

## Conclusions

We have shown evidence to suggest that multimorbidity is now the norm rather than the exception for older Irish adults. The results presented here are based on a novel use of association rules which allow for a fair comparison of the degree of independence of comorbidities. To the author’s knowledge this is the first time such an approach has been taken in the study of multimorbidities^35^. The heterogeneous nature of the combinations of medical conditions reported and the vast differences in their prevalence according to gender highlights the need for the management of these conditions to be considered on a case by case basis and that this management strategy incorporate the entirety of the patient’s physical and mental health rather than treating symptoms of individual medical conditions.

## Methods

### Participants and Data

The data was taken from the third wave of the Irish Longitudinal Study on Ageing (TILDA), and included a population representative sample of 6101 Irish community dwelling adults aged 50+ living across the Republic of Ireland.

### Data

The data studied consisted of the presence of 31 chronic, cardiac and other conditions. These were self-reported diagnoses which were captured during a Computer Assisted Personal Interview (CAPI) at Wave 3 of the TILDA study. The data included in this article includes the life time prevalence of each of these 31 conditions and includes any self reported diagnoses from wave 1,2 or 3 of TILDA which were not later disputed by the respondents. Further details of the participants, the TILDA study design (also previously published^61–63^) and the criteria for deriving binary classifications for some of the medical conditions included in this study can be seen in Supplementary Material S1.

### Statistical methods

Both healthy and multi-morbid participants were included in analysis as the exclusion of healthy subjects would almost certainly have led to over estimation of the significance of disease occurrence by sex as well as the prevalence of individual diseases and their associations with other conditions.

### Univariate analysis

The association between individual morbidities and sex was investigated using a Chi-squared test for significance and were corrected for multiple testing using the false discovery rate^64^. Findings at corrected p-values < 0.05 were considered significant.

### Association rules

Association rules normally take the form *A* = > B which can be read *A* implies *B* for any sets of variables *A* and *B*. In this instance association rules can be used to determine the prevalence and strength of the association between combinations of morbidities which occur more frequently than would be expected by random chance. As the number of possible combinations of associations between variables is vast (2^31^ in this case) the sets *A* and *B* are restricted to be just a single variable here. Three main measurements are used to determine how “interesting” or useful a rule is. They are as follows:

- **Support:** The support is defined as *P*(*A*, *B*) (read the probability of A and B),  or in other words the prevalence of both *A* and *B* co-occurring.

- **Confidence:** The confidence is *P*(*B*|*A*) or the probability that *B* occurs given that *A* is already present.

- **Lift:** The lift (also known as the observed/expected ratio) is measured as $\frac{P(A,B)}{P(A)P(B)}$. The lift is a measure of the degree of dependence between *A* and *B*. A lift of 1 indicates that *A* and *B* are independent and therefore have no association with each other.

### Novel use of association rules

A common approach to prune an interpretable subset of interesting associations is to look at rules which have high support, high confidence and high lift and decide on minimum thresholds for these three values. We argue that interactions and connections of low prevalence diseases may be of great interest and therefore do not impose a lower limit on the support.

Another commonly used approach to mining interesting association rules is to only include rules which have a lift value larger than a given threshold; say 2. This then has the interpretation that only sets of items which co-occur at least twice as often as expected if randomly associated are considered. One issue with such an approach was identified by^35^ who point out that the theoretical upper and lower limits on the range of values the lift can take for a given rule vary. The idea of using a single value threshold for the lift is therefore problematic as the interpretation of a lift value of say 2 which has can range from for example (0,4) will be very different to that of a lift value of 2 that can range from (0,40). To overcome this issue we use the standardised lift described in^35^ which standardises all lift values on a scale of (0,1) using Eq. 1. This allows for direct comparison about the degree of dependence between the antecedent and consequent.

1 $${\rm{Std}}\,{\rm{Lift}}(A=\, > B)=\frac{Lift(A=\, > B)-\frac{{\rm{\max }}\,\{P(A)+P(B)-1,1\,/\,n\}}{(P(A)P(B))}}{\frac{1}{P(A)P(B)}-\frac{{\rm{\max }}\,\{P(A)+P(B)-1,1\,/\,n\}}{(P(A)P(B))}}$$

To the authors’ knowledge this is the first time that such an approach has been taken in the study of comorbidities. All morbidity associations with a standardised Lift of >0.2 were then included for consideration in the analysis of Section 3. A range of values for the threshold of the standardised lift were evaluated and results were not sensitive to the threshold value chosen. More information on the standardised lift along with an illustrative example of its importance can be seen in the Supplementary Material S2.

### Network graphs

Pairwise associations between morbidities were visualised using network graphs. A network graph consists of nodes (morbidities) which are connected by edges (a straight line in the resulting diagram). The edge weight i.e. the thickness of the line connecting any two morbidities (known as the edge) in the network graphs shown in Section 3 is proportional to the number of participants who had the comorbidities signalled by both ends of the edge. The size of the nodes are proportional to the overall prevalence of the indicated condition.

All statistical analysis was conducted using the R statistical software version 3.4.1.^65^.

### Ethics approval and informed consent to participate

Participants were drawn from the third wave of the Irish Longitudinal Study on Ageing (TILDA), a population representative sample of individuals aged 50 and over from across the Republic of Ireland. Details of the sampling design have been provided in a previous study (Whelan and Savva, 2013). The study was approved by the Trinity College Faculty of Health Sciences Ethics Committee, and testing protocols conformed with the Declaration of Helsinki. All participants provided written, informed consent when they first participated in the study (at wave 1) and consent was repeated at wave 3 (the focus of this study), both written and verbal. Respondents in all cases were provided with copies of their signed consent forms.

## Supplementary information

Investigation of multimorbidity and prevalent disease combinations in older Irish adults using network analysis and association rules. Supplementary Information

## Data Availability

An anonymised publicly accessible version of the data that support the findings of this study can be made available on completion of a request form (see https://tilda.tcd.ie/data/accessing-data/). The data are not publicly available in their raw form due to them containing information that could compromise research participant privacy/consent.
