# Supplementary material for: Investigation of multimorbidity and prevalent disease combinations in older Irish adults using network analysis and association rules
Source: Sci Rep. 2019 Oct 10;9:14567. doi: 10.1038/s41598-019-51135-7 (PMC6787335; doi:10.1038/s41598-019-51135-7)
Supplement: Supplementary file 1 — Investigation of multimorbidity and prevalent disease combinations in older Irish adults using network analysis and association rules. Supplementary Information [file 41598_2019_51135_MOESM1_ESM.docx]

# Supplementary Material

# Investigation of multimorbidity and prevalent disease combinations in older Irish adults using network analysis and association rules.

Belinda Hernández*^1,2,3,5^, Richard B. Reilly^1,3,4,5^, Rose Anne Kenny^1,2,3^

*^1^ TILDA The Irish Longitudinal Study in Ageing, Trinity College, The University of Dublin, Ireland*

*^2^Mercer Institute for Successful Ageing, St. James Hospital, Dublin, Ireland*

*^3^ Dept of Medical Gerontology, School of Medicine, Trinity College, The University of Dublin, Ireland*

*^4^ School of Engineering, Trinity College, The University of Dublin, Ireland*

*^5^ Trinity Centre for Biomedical Engineering, Trinity College, The University of Dublin, Ireland*

**Corresponding Author:**

Belinda Hernández, [hernandb@tcd.ie](mailto:hernandb@tcd.ie)

The Irish Longitudinal Study for Ageing,

Trinity College, The University of Dublin, Ireland

# S 1

## Participants

Wave 3 data was collected in 2014 and details of the sampling design have been discussed elsewhere [27]. Data collection was conducted by trained interviewers using Computer Assisted Personal Interviewing (CAPI), and by a self-completion questionnaire (SCQ) which was returned after the interview. All participants were subject to CAPI interviews and were also asked to complete the SCQ. The response rate for wave 3 CAPI and SCQ was 85% (1).

The study was approved by the Trinity College Faculty of Health Sciences Ethics Committee, and testing protocols conformed to the Declaration of Helsinki. All participants provided written, informed consent when they first participated in the study (at wave 1) and consent was repeated at wave 3 (the focus of this study). Respondents in all cases were provided with copies of their signed consent forms.

## Data

The 31 medical conditions included in this study were self-reported diagnoses from the TILDA cohort of participants The life time prevalence of 31 medical conditions was included in this analysis and was estimated using data from waves 1,2, and 3 of the TILDA study. In each wave participants answered questions of the form “Has a doctor ever diagnosed you with X”, with the exception of the conditions mentioned below binary classifications were derived as “Yes” if a participant ever reported a medical diagnoses of a given disease in wave 1,2 or 3 of TILDA and didn’t later dispute this diagnosis.

For further other conditions binary classifications were derived to estimate the life time prevalence:

- Depression was measured and assessed using the Center for Epidemiological Studies Depression Scale (CES-D) as described in (2). The 8 item CES-D-8 was used to screen for depression at waves 1 and 3 where a score of ≥9 on this scale was taken to define cases of depression at wave 3. Wave 1 used a long form of the CES-D scale and so a score of ≥16 indicated depression at wave 1.
- Cases of hearing and vision loss were identified by participants reporting they had either “Poor” or “Very Poor” hearing/vision out of a 5 point scale from ranging from “Excellent” to “Very Poor” or if they were identified as “Registered Blind” in the case of poor vision.
- Obesity was taken as a BMI >=30 where height and weight were based on self-reported measurements from the TILDA Wave 3 CAPI.
- Urinary incontinence was defined as any involuntary loss of urine from the bladder within the last 12 months, based on the International Continence Society Definition (3). Participants were asked ‘During the last 12 months, have you lost any amount of urine beyond your control?’ Those that said yes were defined as having UI.

Table S.1 shows the demographic information for the participants included in this study.

Table 1: Demographics of data studied from wave 3 TILDA

| Variable Name | Number respondents |
| --- | --- |
| Sex:  Male  Female | 2,754  3,347 |
| Age:  50-64  65-75  75+ | 2,846  1,992  1,263 |
| Education:  Primary  Secondary  Third Level | 1,530  2,442  2,128 |
| Marital Status:  Married/Living as if married  Single (never married)  Separated  Divorced  Widowed | 4,269  505  256  193  878 |

# S2: Standarised Lift

McNicolas et al (4) identified that the theoretical upper and lower bounds for the lift, change from rule to rule and are governed by a number of criteria such as the minimum support and confidence threshold placed on the analysis as well as the support of the antecedent and consequent of the rule i.e. the prevalence of the comorbidities in this case. Put simply, a value of 2 on a scale that can range from (0, 4) has a completely different interpretation to a value of 2 which can range from (0, 20). For this reason it may not be a good approach to compare all rules to a lift threshold of one value. Instead (4) suggest that the range of theoretical values the lift can take for each rule should be standardised to so that all standardised lift values take a value between 0 and 1 but their relative distance to their upper and lower bounds remains the same. As all the standardised lift values across different rules are converted to the same scale, this then allows for a fair and direct comparison of the lift across different rules.

Take as an example the following two association rules from the data described in Section 2.2:

Table S2: Example Association Rules and interestingness measures

| **Rule** | **Support** | **Confidence** | **Lift** | **Lift Lower Bound** | **Lift Upper Bound** | **Standardised Lift** |
| --- | --- | --- | --- | --- | --- | --- |
| Lung Disease => Asthma | 0.01 | 0.34 | 4.1 | 1.19 | 11.95 | 0.27 |
| Diabetes => Hypertension | 0.05 | 0.60 | 1.63 | 0.27 | 2.73 | 0.55 |

Focusing only on the first four columns of Table 1, imagine a threshold of 2 was placed on the lift. This would mean only the first association “Lung Disease => Asthma” would be considered in the analysis as its lift value is 4.1. Therefore this rule would be interpreted as Lung Disease and Asthma co-occur 1% of the time (support), the probability of having asthma given that lung disease is already present is 0.34 (confidence) and these two comorbidities co-occur 4.1 times more often than would be expected by random chance (lift). The second rule Diabetes => Hypertension would then be discarded as uninteresting in this case as its lift is less than the threshold 2.

The last three columns show the theoretical upper and lower bounds on the lift as well as the standardised lift using the formulae as described in (4). Here it can be seen that although the lift for the first rule in Table 1 is larger than that of the second, the range of values it can take is also much larger (ranging from 1.19-11.95 as opposed to 0.27-2.73). Taking this information into account it can be seen that actually the second rule may be of more interest as its lift value lies 55% of the way between the theoretical lower and upper bounds of its range as opposed to only 27% of the way for rule 1. For this reason it was decided to use the standardised lift to rank the importance of associations between comorbidities in the following analysis. In all cases a standardised lift <0.2 was discarded from consideration and remaining rules were ranked according to their confidence.

S3 Confidence Measures for all comorbidities


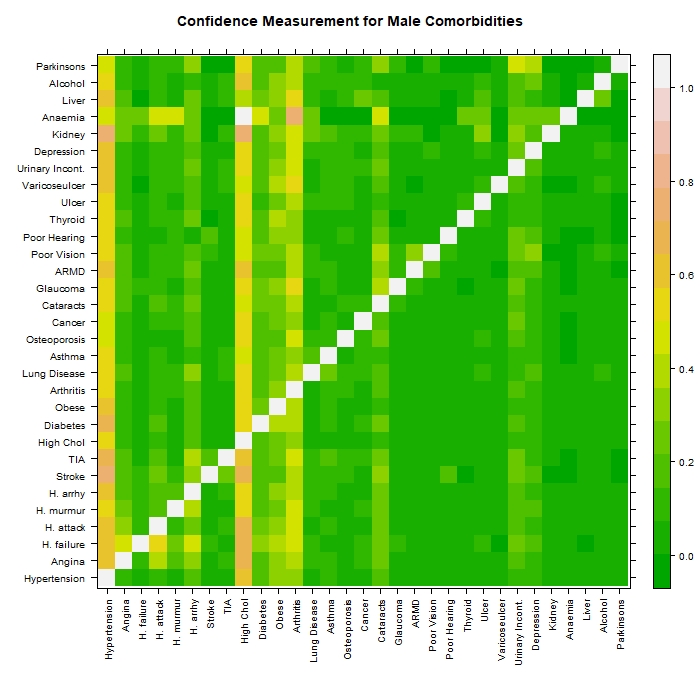


Figure S3.1: Heat map of confidence measures for all posisble comorbidities for males. Index disease is referred to by the y-axis. The grid can be interpreted as the probability of having the morbidity signalled by the x-axis given that a respondent already suffers from the index morbidity signalled by the y-axis


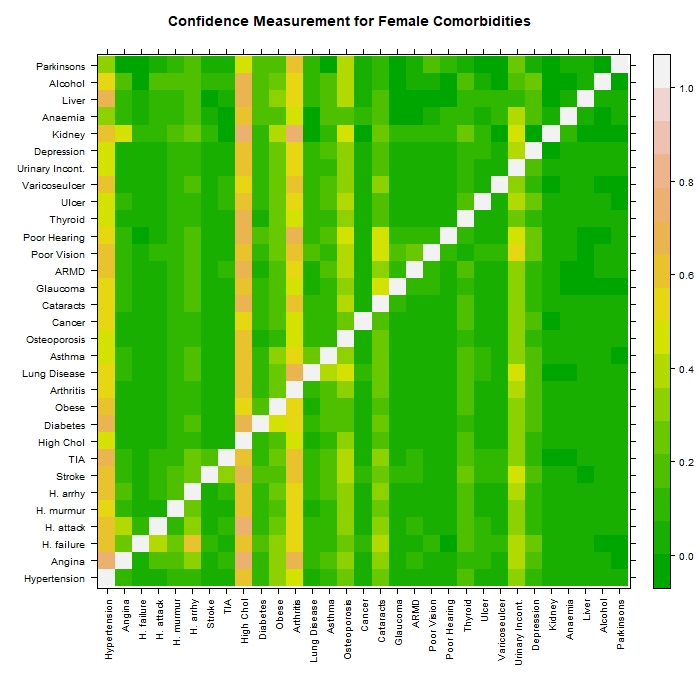


Figure S3.2: Heat map of confidence measures for all posisble comorbidities for females. Index disease is referred to by the y-axis. The grid can be interpreted as the probability of having the morbidity signalled by the x-axis given that a respondent already suffers from the index morbidity signalled by the y-axis

References

1. Donoghue OA, McGarrigle CA, Foley M, Fagan A, Meaney J, Kenny RA. Cohort Profile Update: The Irish Longitudinal Study on Ageing (TILDA). Int J Epidemiol. 2018;47(5).

2. Radloff LS. The CES-D Scale: A Self-Report Depression Scale for Research in the General Population. Appl Psychol Meas. 1977;1(3):385–401.

3. Ulmsten U, Griffiths D, Victor A, Fall M, Abrams P, Rosier P, et al. The standardisation of terminology in lower urinary tract function: report from the standardisation sub-committee of the International Continence Society. Urology. 2003;61(1):37–49.

4. McNicholas PD, Murphy TB, O’Regan M. Standardising the lift of an association rule. Comput Stat Data Anal. 2008;52(10):4712–21.
